# Global disparities in caesarean section rates: Why indication-based metrics are needed

**DOI:** 10.1371/journal.pgph.0002877

**Published:** 2024-02-06

**Authors:** Mehreen Zaigham, John Varallo, Shakila Thangaratinam, Wanda Nicholson, Gerard H. A. Visser

**Affiliations:** 1 Obstetrics and Gynaecology, Institution of Clinical Sciences Lund, Lund University, Lund, Sweden; 2 Global Surgery Foundation, Geneva, Switzerland; 3 WHO Collaborating Centre for Global Women’s Health, Institute of Metabolism and Systems Research, University of Birmingham, Birmingham, United Kingdom; 4 Birmingham Women’s and Children’s NHS Foundation Trust, Birmingham, United Kingdom; 5 Department of Prevention and Community Health, George Washington Milken Institute of Public Health and the Department of Obstetrics and Gynaecology, George Washington School of Medicine, Washington, DC, United States of America; 6 Department of Obstetrics, University Medical Center, Utrecht, The Netherlands; Aga Khan University Medical College Pakistan, PAKISTAN

As stakeholders and maternal healthcare leaders gathered for the XXIV FIGO World Congress of Gynaecology and Obstetrics in Paris France, disparities in access to safe and timely caesarean birth were high on the agenda. Cesarean section is a life-saving intervention for mothers and babies and one of the most common surgical procedures performed globally [1]. With an estimated 38 million procedures, it is projected that every third child will be born by caesarean section by 2030 Fig 1 [2]. A timely operation can prevent maternal-neonatal deaths and the underuse of caesarean section in such cases can contribute to increased mortality and morbidity. Conversely, the overuse of the operation without a clear clinical indication has no medical benefit to the mother or newborn and can negatively impact maternal-child health and waste valuable healthcare resources [3, 4]. There is emerging evidence that babies born by caesarean section have different hormonal, physical, bacterial, and medical exposures affecting their immunological development as compared to vaginally born babies [3, 5].

**Fig 1:**
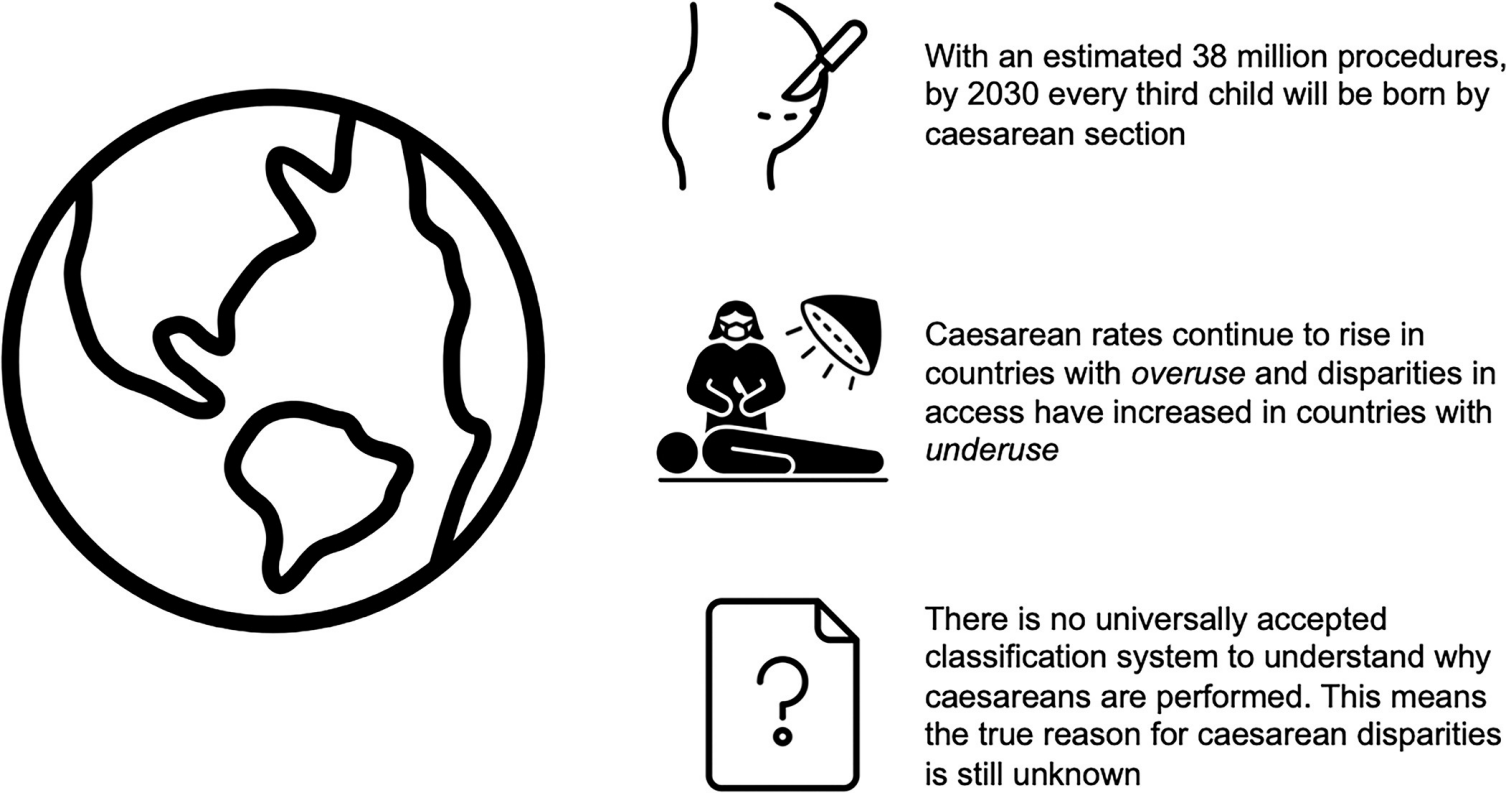
Global disparities in caesarean sections are widening, with no clear understanding of why rates are increasing so dramatically.

Across and within countries, there are tremendous disparities in caesarean section rates. From over 63.4% in Eastern Asia to 7.1% in Sub-Saharan Africa, optimising caesarean section rates is a global crisis and must be a priority for public health leaders and clinicians [2]. Population-based estimates mask equity differences within countries and amongst population groups that either get too few (underuse) or too many operations (overuse) [2]. Despite much research, little impact has been made to correct the inequitable distribution of caesarean sections and to align rates with the indication for surgical birth. In the last twenty years, rates have continued to rise in countries with overuse and disparities in access have increased in countries with underuse [2]. Importantly, healthcare access is not synonymous to caesarean access, as operations may be higher in facilities where healthcare access is poor or delayed and in women who present with more unmanaged complications. For example, in many low- and middle-income countries, vaginal instrumental births are limited or absent. This may well indicate that health care personnel that were taught to perform caesarean sections may do so because of insufficient vaginal birth skills [6]. Adding further complexity to the growing problem of overuse, in a review by Kingdon et al. [7] inter- and intra-system power differentials and differing stakeholder commitment strongly effected caesarean section rates independent of the efficacy of targeted interventions to reduce them.

Current efforts in caesarean section classification have utilised the Robson Classification system [8], also known as the ‘ten-group classification’, as a way to assess, monitor, and compare caesarean section rates within and across healthcare systems. The Robson classification system gained popularity due to its clearly defined, easily reproducible and mutually exclusive categories [9]. However, this system identifies the ‘**whom**’ (obstetric or maternal characteristics) but not the ‘**why’** (clinical reasons for the operation which may or may not be medically indicated). The significant heterogenicity of the caesarean section group makes it particularly challenging to categorise and make valid comparisons across settings. Caesarean sections have a multitude of reasons: clinical (maternal/foetal), women and communities, health professionals, and organizational and system factors [10]. Understanding the reasons behind these global disparities is key. Are rates ‘doctor’ or ‘women’ driven? Are there monetary incentives for doctors or hospitals? Or is it convenience, fear of litigation, inappropriate knowledge of foetal monitoring and birthing techniques on the one hand, and/or fear of childbirth, inadequate information on risks and benefits of caesarean sections on the other hand?

In 2011, a systematic review by Torloni and colleagues identified 27 different systems for classifying caesarean sections [11]. The authors compared the advantages and deficiencies of each system using feedback from a questionnaire with 12 case-scenarios retrieved from a panel of 38 international experts. Their results suggested that a women-based classification system, and the Robson’s in particular, was most suitable to the characteristics required by the international expert group [11]. In a 2015 statement by the World Health Organization (WHO), the lack of a standardised internationally accepted classification system to monitor and compare caesarean section rates was mentioned as one of the key factors hindering a better understanding of operative trends [12]. The WHO proposed adopting the Robson’s classification. However, almost a decade on from this recommendation, policymakers are still looking for answers to why caesarean section trends vary so drastically from region to region. Professional organizations have made recommendations to modify the Robson’s with criteria enabling comparison of caesarean section rates and indications [13].

Currently, there is no universally accepted classification system to understand **‘why’** caesareans are performed, and with variations in reporting of operative indications, this means the true reason for caesarean section disparities is still unknown (Fig 1). The UK Medical Research Council funded C-Safe Programme [14], which aims to reduce unnecessary caesarean sections and make operations safe, is currently addressing the lack of a robust classification system for caesarean sections through its C-Why component. The C-Why brings together clinicians, researchers, policymakers and women and communities to develop a standardised, evidence-based, clinically relevant system for reporting caesarean indications. Such a study is important and should also be carried out in other parts of the world. Special emphasis should be given to possible discrepancies between indications according to the doctor and those perceived by the women.

To decrease disparities in global caesarean section rates, it is essential to identify not only what groups of women are undergoing the operation but to also pinpoint the underlying reasons for caesareans across settings. This is currently lacking and there is a dire need for indication-based metrics to decipher differences in global caesarean rates. There can be merit in combining the existing Robson’s classification with an additional metric examining the **‘why’** component for caesarean sections. For example, a facility-based study by Abubeker and colleagues [15] in a tertiary hospital in Ethiopia, found high rates of caesarean section in low-risk women identified using the Robson classification. As a next step, it would have been immensely valuable for healthcare authorities to extract the actual reasons for the drivers in caesarean sections in these women. In essence, the **‘whom’** and the **‘why’** are both essential for us to truly grasp the reasons driving variations in caesarean section rates across the world. Only then can global health experts and policy-makers formulate and execute targeted strategies and interventions to increase resources for safe operative birth in settings of ‘underuse’ and the employment of mitigation strategies for control in settings of ‘overuse’. This, in turn, may have a major impact in accelerating ongoing efforts for improving maternal and perinatal outcomes globally.
